# Weight-Adjusted-Waist Index Predicts Newly Diagnosed Diabetes in Chinese Rural Adults

**DOI:** 10.3390/jcm12041620

**Published:** 2023-02-17

**Authors:** Shasha Yu, Bo Wang, Xiaofan Guo, Guangxiao Li, Hongmei Yang, Yingxian Sun

**Affiliations:** 1 Department of Cardiology, First Hospital of China Medical University, 155 Nanjing North Street, Heping District, Shenyang 110001, China; 2 Department of Clinical Epidemiology, Institute of Cardiovascular Diseases, First Hospital of China Medical University, Shenyang 110001, China

**Keywords:** weight-adjusted waist index, diabetes, obesity, rural

## Abstract

The relationship between the weight-adjusted waist index (WWI) and newly diagnosed type 2 diabetes (T2D) remains uncertain. This study intended to explore the association between the WWI and the incidence of newly diagnosed T2D among participants in rural areas of China. In the Northeast China Rural Cardiovascular Health Study, 9205 non-diabetic individuals (mean age 53 ± 10, 53.1% women) without T2D were included at baseline during 2012–2013. They were followed up from 2015 to 2017. WWI was calculated as waist circumference (cm) divided by the square root of weight (kg). We used multivariate logistic regression models to estimate odds ratios (ORs) and 95% confidence intervals (CIs) for the probability of new diagnoses across three WWI categories. A total of 358 participants had been diagnosed with T2D during a median follow-up of 4.6 years. After adjusting for potential confounders, compared with the lowest WWI category (<9.79 cm/√kg in men; <10.06 in women), men with WWI 10.06–10.72 and ≥10.37 cm/√kg showed OR (95%CI) for T2D 1.200 (0.816, 1.767) and 1.604 (1.088, 2.364), respectively, while women with WWI 10.06–10.72 and ≥10.37 cm/√kg showed ORs (95%CIs) for T2D 1.191 (0.703, 2.018) and 1.604 (1.088, 2.364), respectively. The ORs were generally consistent on subgroup analysis by gender, age, body mass index, and current smoking and drinking status. Increasing WWI was significantly associated with a higher incidence of newly diagnosed T2D among rural Chinese adults. Our findings help clarify the harmful effect of increasing WWI on newly diagnosed T2D and provide evidence for formulating healthcare policy in rural China.

## 1. Introduction

The estimated prevalence of type 2 diabetes (T2D) in adults aged 20 to 79 worldwide in 2021 was 10.5% (536.6 million people), and it is anticipated to increase to 12.2% (783.2 million people) in 2045 [1]. T2D was anticipated to be more prevalent in high-income countries compared with low-income countries and becomes a huge health burden in low- and middle-income countries [2]. Compared with high-income (12.2%) and low-income (11.9%) countries, middle-income countries are predicted to experience the largest relative increase in T2D prevalence between 2021 and 2045 (21.1%) [1]. Global economic development is still heavily impacted by the prevention and treatment of T2D, which also endangers the lives and health of patients. In particular, T2D is an important public health problem in China [3]. Despite improvements, T2D awareness and management are still generally poor [4]. Therefore, in order to prevent T2D, improve care, and reduce the number of diabetes-related early deaths, particularly in China, it is vital to identify modifiable risk factors.

The age-standardized prevalence of central obesity and general obesity increased from 15.8% and 0.2%, respectively, in 1993 to 30.3% and 0.9%, respectively, in 2011. In 2011, the proportion of rural individuals with central obesity was higher than the proportion of urban adults (29.6% vs. 30.6%, compared with 20.8% vs. 13.4% in 1993) [5]. In the last ten years, obesity in rural China has become a serious health concern. Given the connection between obesity and T2D, strict weight control may help prevent T2D [6]. Accordingly, increasing studies have explored the connection between new obesity indicators, T2D, and other metabolic disorders. Many anthropometric measurements have been suggested as indicators to identify individuals with a high risk of T2D [7]. A novel adiposity index named the weight-adjusted waist index (WWI) was proposed in 2018 as a new obesity anthropometric index [8]. Unlike BMI, which cannot distinguish between fat and muscle mass, WWI can better distinguish fat and muscle mass components and mainly reflects the problem of central obesity independent of body weight [9]. Cumulative evidence confirmed that the increasing value of WWI can be a useful risk factor for hyperuricemia, left ventricular hypertrophy, urinary albumin excretion, and abdominal aortic calcification [10,11,12,13]. However, the effect of WWI on the incidence of T2D has not been investigated before in rural Chinese patients. To improve treatment results and take into account the higher risk of diabetes-related adverse events in this high-risk demographic, it is important to pinpoint the relationship between WWI and T2D in rural Chinese people. Therefore, the objective of this study is to evaluate the relationship between WWI and T2D in the rural Chinese population and lay the groundwork for the early detection of T2D patients.

## 2. Methods

### 2.1. Study Design and Participants

**Figure 1:**
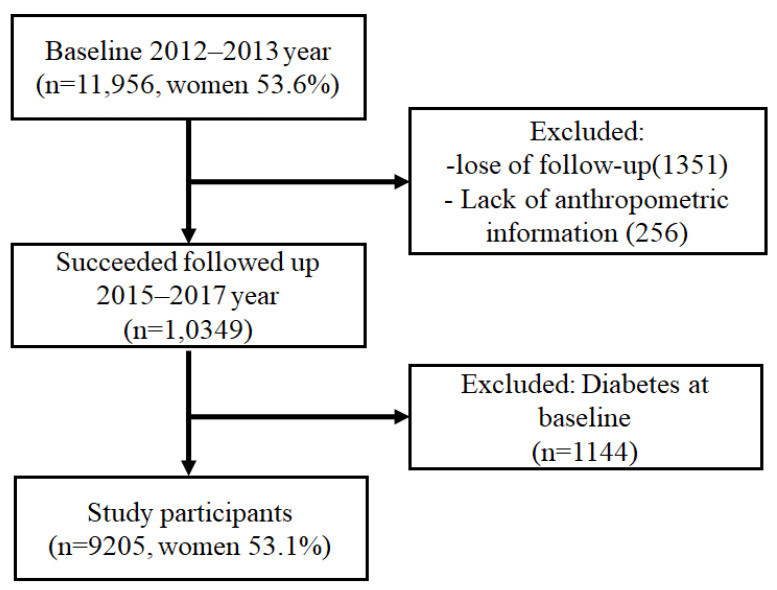
The recruitment process of the study population.

A community-based prospective cohort study called the Northeast China Rural Cardiovascular Health Study (NCRCHS) was conducted in rural Northeast China. The specific sampling techniques and admission requirements for this study have been described previously [14]. Multi-stage, stratified, and random cluster sampling were used for the study’s design. From three directional areas of the Liaoning Province, the counties of Dawa, Zhangwu, and Liaoyang were selected for the first stage of the study. A town from each county was chosen randomly for the following study phase (a total of three towns). The final stage of the study involved randomly selecting 8–10 rural villages from each town, for a total of 26 rural villages. Each village’s permanent residents eligible for this study (35 years of age or older) were sent an invitation to participate. Pregnant women, people with malignant tumors, and people with mental illnesses were excluded from the study [15]. The China Medical University Ethics Committee gave their approval to this work (Shenyang, China, AF-SDP-07-1, 0-01). The baseline information for the study was obtained from the 2012–2013 survey results, and a total of 11,956 participants were finally enrolled. The study cohort was followed up from 2015 to 2017, and the median follow-up period was 4.66 years. The detailed participant inclusion process is outlined in Figure 1. Eventually, the data of 9205 participants were considered for analyses.

### 2.2. Study Variables

In light clothing and without shoes, the subjects’ height and weight were recorded. We used non-elastic tape to measure the waist circumference at the umbilicus. An electronic, standardized, automated manometer (HEM-907; Omron, Tokyo, Japan) was used to measure participants’ blood pressure three times after at least five minutes of rest while seated. Blood was drawn from participants after they had fasted for at least 12 h. Low-density lipoprotein cholesterol (LDL-C), fasting plasma glucose (FPG), and other routinely assessed biochemical indices were determined enzymatically.

**Figure 2:**
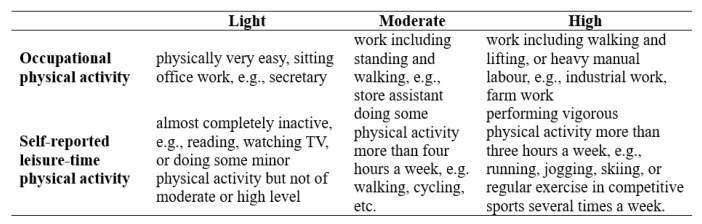
Physical activity categories.

An interview utilizing a standardized questionnaire was conducted at the baseline to gather data on demographic traits, lifestyle factors, and medical history. The current use of tobacco and alcohol was defined. The number of hours of sleep in a 24 h period was used to define total sleep time. Education level was determined by whether a person had completed elementary school, middle school, or high school. Family income was classified as ≤5000 CNY/year (788 dollar/year), 5000–20,000 (788–3152 dollar/year) and >20,000 CNY/year (3152 dollar/year). The participants’ physical activity, including their occupational as well as leisure-time physical activities, was also assessed in the present study [14]. The participants were divided into the following three categories based on their professional and recreational physical activities (Figure 2): (1) low activity: participants who reported low levels of both occupational and leisure-time physical activities; (2) moderate activity: participants who reported moderate or high levels of either occupational or leisure-time physical activity; and (3) high activity: participants who reported moderate or high levels of both occupational and leisure-time physical activities.

### 2.3. Definition

The following equation was used to determine the body mass index (BMI):

$$\frac{weight\;\left(\mathrm{kg}\right)}{{\left(height\right)}^{2}\;\left(m\right)}$$

Patients were divided into three groups based on WWI tertiles. WWI tertiles in men were defined as follows: <9.79 cm/√kg (Q1), ≥9.79 and <10.37 cm/√kg (Q2), and ≥10.37 cm/√kg (Q3). WWI tertiles in women were <10.06 cm/√kg (Q1), ≥10.06 and <10.72 cm/√kg (Q2), and ≥10.72 cm/√kg (Q3).

Diabetes was defined as a fasting plasma glucose (FPG) value ≥ 7.0 mmol/L or a previously diagnosed diabetes [16].

### 2.4. Statistical Analysis

Continuous variables are reported as the mean ± standard deviation, and categorical variables are reported as numbers and percentages. Differences among categories were evaluated using the student’s *t*-test or ANOVA for parametric data and the χ^2^ test for non-parametric data, as needed. Multiple logistic regression models were used to estimate odds ratios (ORs) and 95% confidence intervals (CIs) in order to examine the relationship between the occurrence of T2D and WWI. We developed three models: (1) unadjusted, (2) adjusted for age, current smoking, and drinking, and (3) adjusted for age, heart rate, current smoking, current drinking, HDL-C, LDL-C, BMI, SBP, DBP, sleep duration, educational status, ethnicity, annual income (CNY/year), and physical activity at baseline. Subgroup analyses were stratified by sex (men or women), age (<60 or ≥60 years), BMI (<24 or ≥24 kg/m^2^), current smoking (yes or no), and current drinking (yes or no). Statistical analyses were performed using the SPSS version 20.0 software (SPSS Inc., Chicago, IL, USA), and *p* < 0.05 was considered statistically significant.

## 3. Results

### 3.1. Clinical Characteristics of the Study Population

Table 1 and Table 2 display the clinical and demographic details of the study population according to the men’s and women’s WWI categories. Compared with the participants in the lowest WWI category (<9.79 cm/√kg in men; <10.06 cm/√kg in women), those in the higher WWI category had higher values of age, BMI, WC, systolic blood pressure (SBP), diastolic blood pressure (DBP), LDL-C, and FPG, whereas they had lower values of height and HDL-C. As shown in Table 1, from Q1 to Q3, the rate of current smoking was lower in the higher WWI category. In contrast, current drinking, low educational status, lower annual income, longer sleep duration, and low physical activity were more prevalent in higher WWI categories. Table 2 shows that from Q1 to Q3, the rate of current smoking increased, and lower educational status, lower annual income, longer sleep duration, and low physical activity became more prevalent.

### 3.2. Association between WWI and Newly Diagnosed T2D

The correlation between WWI and the risk of T2D is shown in Table 3. The univariate analysis (Model 1) revealed that WWI was associated with an increased risk of T2D. After adjustment for age, current smoking, and drinking (Model 2) and further adjustment for age, heart rate, current smoking, current drinking, HDL-C, LDL-C, BMI, SBP, DBP, sleep duration, educational status, ethnicity, annual income (CNY/year), and physical activity (Model 3), WWI and T2D remained independently associated. Compared with the lowest WWI category or Q1 (<9.79 cm/√kg in men; <10.06 cm/√kg in women), the probability of T2D significantly increased [OR (95%CI) = 1.604 (1.088, 2.364) for men; OR (95%CI) = 1.899 (1.121, 3.218) for women] in Q3 (≥10.37 cm/√kg in men; ≥10.72 cm/√kg in women). In addition, the incidence of T2D increased with increasing WWI tertiles in all models (P for trend < 0.05).

### 3.3. Subgroup Analyses for Association between WWI and T2D

**Figure 3:**
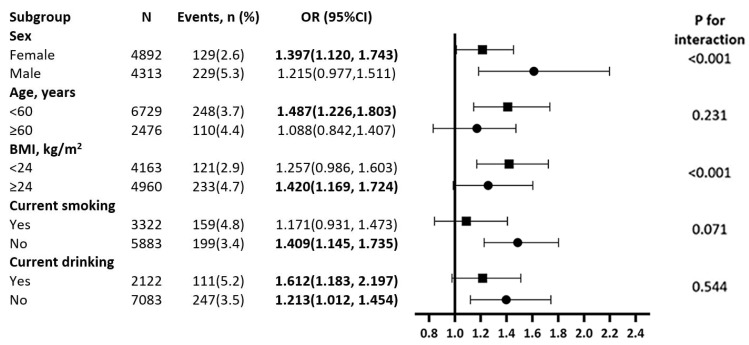
Subgroup analysis of the association between WWI and incidence of diabetes among rural Chinese. Each subgroup analysis is adjusted if not stratified for age, heart rate, current smoking, current drinking, high density lipoprotein, low density lipoprotein, body mass index, systolic blood pressure, diastolic blood pressure, sleep duration, educational status, ethnicity, annual income (CNY/year), and physical activity.

The subgroups of sex, age, BMI, current smoking, and current drinking were used to assess the relationship between the highest WWI category, or Q3 (10.37 cm/kg in men and 10.72 cm/kg in women), and the risk of T2D while using the lowest WWI category, or Q1 (9.79 cm/kg in men and 10.06 cm/kg in women), as the reference. After adjusting for all factors with the exception of the stratified variable, the relationship between WWI and T2D held true in all subgroup analyses (Figure 3). We also performed an interaction analysis of the WWI tertiles with each stratification factor, but we could not find any interactions between them other than with age and BMI.

## 4. Discussion

A longitudinal population-based study was conducted to investigate the association between the WWI, a new adiposity index, and T2D in rural Chinese participants. Men and women in the highest WWI category (≥10.37 cm/√kg in men; ≥10.72 cm/√kg in women) had a 1.604-fold and 1.899-fold, respectively, increased risk of T2D as compared with those in the lowest WWI category (<9.79 cm/√kg in men; <10.06 cm/√kg in women). For the first time, our study confirmed that the incidence of T2D increased with WWI tertials and that there was a significant correlation between WWI and T2D. This correlation was not influenced by characteristics such as sex, age, lifestyle, BMI, WC, lipid profile, or other cardiovascular or cerebrovascular risk factors.

Cumulative evidence confirms that obesity, characterized by excessive body fat, increases the risk of many chronic diseases such as hypertension, hyperlipidemia, T2D, cardiovascular diseases, and cerebrovascular diseases [17]. Of these, obesity significantly increases the risk for T2D risk; this has been proven by several previous studies using a wide range of anthropometric measures such as BMI, waist circumference, waist-to-height ratio, waist-to-hip ratio, and a body shape index (ABSI) defined by WC/(BMI2/3Height1/2) [17,18,19]. Although several studies concluded the predictive value of BMI for metabolic disorders to be effective, some investigators suggest the BMI as a reliable indicator of obesity is limited in that it is incapable of distinguishing between lean mass and fat mass and its variation due to age, gender, and race difference [20,21]. A similar situation presents itself in the case of other traditional and newer anthropometric indicators. Therefore, it is necessary to identify an integrated adiposity index with the ability to better predict the risk of metabolic disorders to decrease cardiometabolic disease-associated morbidity and mortality. Some nontraditional obesity indicators, such as the visceral adiposity index (VAI), lipid accumulation product (LAP) index, and product of triglycerides and glucose (TyG) have been used to predict T2D risk and shown area under the curve values of 0.687, 0.743, and 0.762, respectively [22]. Wei et al. reported that higher ABSI scores are independently associated with T2D risk [23]. Some of these non-traditional obesity indicators, despite being widely discussed, are difficult to quantify and have limited practical applicability. Others have been highly clustered around the mean with a rather small variance, making it difficult to define a clinical cutoff for clinical practice [24].

As a novel adiposity index, WWI, which is easier to calculate, is an integrated predictor of morbidity which has been linked to cardiometabolic illness since 2018 [19]. To our knowledge, this is the first longitudinal study examining the relationship between WWI and newly diagnosed T2D. WWI has been explored in a wide range of fields, particularly those related to hypertension, albuminuria, hyperuricemia, left ventricular hypertrophy, and abdominal aortic calcification [10,11,12]. The results of these studies reinforce the effectiveness of WWI as a novel indicator of abdominal obesity to predict newly diagnosed T2D in rural China. The potential mechanism of the positive correlation between WWI and T2D can be explained by the role of abdominal fat as a marker of ectopic fat excess. The increase of WWI may reflect the dysfunction of adipose tissue, thereby causing an increase in proinflammatory cytokines and chemokines, immune cell infiltration, an accumulation of senescent cells, and resulting in insulin resistance, chronic sterile inflammation, and lipid redistribution [25]. First, obesity-driven insulin resistance in liver, white adipose tissue (WAT) and skeletal muscle, combined with insufficient secretion of insulin by pancreatic β-cells to overcome this resistance [26]. Second, obesity is closely associated with increased inflammatory markers in the liver, adipose tissue, skeletal muscle, pancreatic islets, and brain. Obesity induced inflammation is directly associated with insulin resistance and finally caused T2D. Many pathways such as NF-κB, JNK, and TNF-α, as well as numerous other proinflammatory signaling molecules, scaffolding proteins, and cytokines, have been proved to take part in this process [27,28,29,30]. Furthermore, WWI can be a useful interventional target to decrease T2D incidence in the rural population. First, in rural areas, as villagers have physical examinations, village doctors calculate the WWI for each villager and record the value. WWI may serve as a simple and effective anthropometric index in clinical practice.

For those with a higher WWI value, village doctors need to pay more attention to their blood glucose level since WWI correlates with a higher risk of T2D. Simultaneously, village doctors conduct healthy knowledge propagation and body weight control education. Our previous study already reported the effectiveness of village doctor-led intervention which resulted in statistically significant improvements in blood pressure control among rural residents in China [31]. Second, village doctors should consider the changes in WWI values to evaluate the effectiveness of body weight control. Once the WWI value is reduced to normal, it represents a decreased risk of getting T2D. Third, according to landmark randomized trials and clinical guidelines, populations with a higher WWI value deserve high priority in diabetic healthcare.

Additionally, our results also suggested that WWI, as a continuous parameter, is positively correlated with T2D incidence for current drinking and non-drinking participants. However, the connection between WWI and T2D incidence among ≥ 60-year-old, BMI ≥ 24 kg/m^2^, and current smoking subjects was no longer significant. Similarly, a prior study found that there was a closer correlation between abdominal obesity and T2D in younger Japanese people than in older Japanese people [32]. This could be explained by the varied body fat distribution between older and younger people [33]. Future investigations using different anthropometric measures for fat distribution may help clarify this phenomenon. In the current investigation, one intriguing finding was that the association between WWI and T2D was not significant among current smokers after adjusting for possible confounders. This was inconsistent with previous studies. According to Luo et al., there is a substantial interaction between current smoking and abdominal obesity on T2D, but there is no statistically significant interaction between smoking and overall obesity on T2D [34]. This discrepancy may be due to the relatively high prevalence of smoking among men (57.9% vs. 16.8%, *p* < 0.001), ≥60-year-old (37.5% vs. 35.6%, *p* = 0.044), and people with a BMI < 24 kg/m^2^ (41.6% vs. 21.6%, *p* < 0.001).

Our study has several strengths, including the large sample size and longitudinal prospective study design. To the best of our knowledge, this study is the first to estimate the relationship between WWI and T2D incidence among the rural Chinese population. In this study, all subjects were free of T2D at baseline; therefore, the relationship between WWI and T2D incidence could be evaluated more precisely. Nevertheless, there are some limitations to the present study. First, the participants came from one province in northeast China, and therefore, the findings cannot be generalized across the country. Second, the incidence of T2D was based on a single blood test and may have been biased. Third, although we adjusted for possible confounders, residual confounding factors may still exist. Fourth, we lost contact with some participants during the follow-up, which might have caused bias in assessing the relationship between WWI and newly diagnosed T2D. Finally, we only evaluated the status of current alcohol drinking; information on the frequency of alcohol drinking, beverage type, and overall average weekly alcohol intake was not obtained. Therefore, there may have been inaccuracies in the description of the drinking status. This could have had an impact on the potential relationship between WWI and T2D.

## 5. Conclusions

We discovered an independent, favorable correlation between the incidence of T2D and WWI in the rural Chinese population. WWI can act as a simple and effective predictor of T2D and may be helpful in preventing T2D in the rural Chinese population.

## Figures and Tables

**Table 1:** Baseline characteristics of study participants by weight-adjusted waist index in male.

Variables	Weight-Adjusted Waist Index (WWI) (cm/√kg)			
	Q1 (<9.79)	Q2 (9.79–10.37)	Q3 (≥10.37)	*p* Value
**N**	1419	1433	1419	
**Age (years)**	51.83 ± 9.87	53.31 ± 10.46	56.79 ± 11.20	<0.001
**Current smoking (yes)**	888 (62.6)	829 (57.9)	758 (53.4)	<0.001
**Current drinking (No)**	615 (43.3)	703 (49.1)	633 (44.6)	0.006
**Ethnicity ^a^ (Han)**	1347 (94.9)	1343 (93.7)	1327 (93.5)	0.229
**Education status**				0.002
Primary school or below	545 (38.4)	571 (39.8)	642 (45.2)	
Middle school	710 (50.0)	677 (47.2)	623 (43.9)	
High school or above	164 (11.6)	185 (12.9)	154 (10.9)	
**Annual income (CNY/year)**				<0.001
≤5000	136 (9.6)	157 (11.0)	245 (17.3)	
5000–20,000	780 (55.0)	774 (54.1)	769 (54.2)	
>20,000	503 (35.4)	499 (34.9)	405 (28.5)	
**Sleep duration (h/d)**				0.027
≤7	676 (47.7)	627 (43.8)	664 (46.8)	
7–8	429 (30.3)	441 (30.8)	387 (27.3)	
8–9	202 (14.3)	241 (16.8)	222 (15.7)	
>9	110 (7.8)	122 (8.5)	145 (10.2)	
**Physical activity**				<0.001
Low	310 (22.0)	365 (25.6)	485 (34.6)	
Moderate	268 (19.0)	279 (19.6)	247 (17.6)	
High	829 (58.9)	781 (54.8)	670 (47.8)	
**Pulse (times/min)**	75 ± 13	75 ± 12	76 ± 13	0.222
**BMI (kg/m^2^)**	22.92 ± 2.68	24.90 ± 3.13	26.08 ± 3.79	<0.001
**WC (cm)**	74.95 ± 6.17	83.76 ± 6.64	90.97 ± 7.92	<0.001
**Height (m)**	167.68 ± 5.71	166.77 ± 6.37	164.89 ± 6.68	<0.001
**SBP (mmHg)**	136.61 ± 19.19	142.73 ± 21.97	149.34 ± 23.32	<0.001
**DBP (mmHg)**	80.90 ± 10.87	83.78 ± 11.89	85.79 ± 11.81	<0.001
**LDL-C(mmol/L)**	2.73 ± 0.74	2.89 ± 0.78	3.02 ± 0.81	<0.001
**HDL-C (mmol/L)**	1.51 ± 0.42	1.42 ± 0.46	1.36 ± 0.41	<0.001
**FPG (mmol/L)**	5.50 ± 0.53	5.58 ± 0.55	5.59 ± 0.58	<0.001

^a^ others including some ethnic minorities in China, such as Mongol and Manchu. Abbreviations: CNY China Yuan (1 CNY = 0.161 USD), BMI body mass index, WC waist circumference, SBP Systolic Blood pressure, DBP diastolic blood pressure, FPG fasting plasma glucose, HDL-C high-density lipoprotein, LDL-C low-density lipoprotein cholesterol.

**Table 2:** Baseline characteristics of study participants by weight-adjusted waist index in female.

Variables	Weight-Adjusted Waist Index (WWI) (cm/√kg)			
	Q1 (<10.06)	Q2 (10.06–10.72)	Q3 (≥10.37)	*p* Value
**N**	1618	1622	1608	
**Age (years)**	48.83 ± 9.00	51.74 ± 9.13	57.61 ± 10.45	<0.001
**Current smoking (yes)**	245 (15.1)	270 (16.6)	306 (19.0)	0.012
**Current drinking (No)**	43 (2.7)	45 (2.8)	63 (3.9)	0.075
**Ethnicity ^a^ (Han)**	1535 (94.9)	1528 (94.2)	1505 (93.6)	0.299
**Education status**				<0.001
Primary school or below	705 (43.6)	909 (56.0)	1099 (68.3)	
Middle school	732 (45.2)	576 (35.5)	436 (27.1)	
High school or above	181 (11.2)	137 (8.4)	73 (4.5)	
**Annual income (CNY/year)**				<0.001
≤5000	106 (6.6)	144 (8.9)	264 (16.4)	
5000–20,000	866 (53.5)	913 (56.2)	935 (58.1)	
>20,000	646 (39.9)	564 (34.8)	409 (25.4)	
**Sleep duration (h/d)**				<0.001
≤7	857 (53.0)	834 (51.5)	841 (52.4)	
7–8	485 (30.0)	479 (29.1)	395 (24.6)	
8–9	175 (10.8)	196 (12.1)	242 (15.1)	
>9	99 (6.1)	111 (6.9)	128 (8.0)	
**Physical activity**				0.001
Low	598 (37.4)	618 (38.3)	709 (44.5)	
Moderate	323 (20.2)	309 (19.2)	285 (17.9)	
High	680 (42.5)	686 (42.5)	601 (37.7)	
**Pulse (times/min)**	79 ± 12	79 ± 13	79 ± 13	0.331
**BMI (kg/m^2^)**	23.28 ± 3.40	24.95 ± 3.36	25.93 ± 4.12	<0.001
**WC (cm)**	72.61 ± 6.73	80.82 ± 6.76	88.06 ± 8.44	<0.001
**Height (m)**	157.31 ± 5.62	156.12 ± 5.61	153.56 ± 6.48	<0.001
**SBP (mmHg)**	132.00 ± 20.19	138.97 ± 22.71	145.82 ± 25.29	<0.001
**DBP (mmHg)**	77.98 ± 10.44	80.71 ± 11.25	81.91 ± 11.73	<0.001
**LDL-C(mmol/L)**	2.77 ± 0.78	2.97 ± 0.82	3.13 ± 0.87	<0.001
**HDL-C (mmol/L)**	1.47 ± 0.34	1.41 ± 0.33	1.41 ± 0.36	<0.001
**FPG (mmol/L)**	5.39 ± 0.52	5.44 ± 0.55	5.53 ± 0.55	<0.001

^a^ others including some ethnic minorities in China, such as Mongol and Manchu. Abbreviations: CNY China Yuan (1 CNY = 0.161 USD), BMI body mass index, WC waist circumference, SBP Systolic Blood pressure, DBP diastolic blood pressure, FPG fasting plasma glucose, HDL-C high-density lipoprotein, LDL-C low-density lipoprotein cholesterol.

**Table 3:** Logistic regression analysis for the association between newly diagnosed diabetes and weight-adjusted waist index.

WWI (cm/√kg)	Model 1		Model 2		Model 3	
Male	OR (95%CI)	*p* Value	OR (95%CI)	*p* Value	OR (95%CI)	*p* Value
**Q1 (<9.79)**	1		1		1	
**Q2 (9.79–10.37)**	1.398 (0.968, 2.019)	0.074	1.377 (0.952, 1.991)	0.089	1.200 (0.816, 1.767)	0.354
**Q3 (≥10.37)**	2.121 (1.505, 2.991)	<0.001	2.047 (1.442, 2.905)	<0.001	1.604 (1.088, 2.364)	0.013
**P for trend**	<0.001		<0.001		0.013	
**Female**						
**Q1 (<10.06)**	1		1		1	
**Q2 (10.06–10.72)**	1.487 (0.891, 2.482)	0.129	1.468 (0.878, 2.457)	0.144	1.191 (0.703, 2.018)	0.516
**Q3 (≥10.72)**	2.727 (1.712, 4.344)	<0.001	2.642 (1.616, 4.319)	<0.001	1.899 (1.121, 3.218)	0.017
**P for trend**	<0.001		<0.001		0.011	

Model 1: Unadjusted; Model 2: Adjusted for age, current smoking and drinking; Model 3 Adjusted for age, heart rate, current smoking, current drinking, high density lipoprotein, low density lipoprotein, body mass index, systolic blood pressure, diastolic blood pressure, sleep duration, educational status, ethnicity, annual income (CNY/year), physical activity.

## Data Availability

Data can be provided by the corresponding author upon reasonable request.

## References

[1] Sun H., Saeedi P., Karuranga S., Pinkepank M., Ogurtsova K., Duncan B.B., Stein C., Basit A., Chan J.C.N., Mbanya J.C. (2022). IDF Diabetes Atlas: Global, regional and country-level diabetes prevalence estimates for 2021 and projections for 2045. Diabetes Res. Clin. Pract..

[2] Zhang P., Gregg E. (2017). Global economic burden of diabetes and its implications. Lancet Diabetes Endocrinol..

[3] Liu M., Liu S.W., Wang L.J., Bai Y.M., Zeng X.Y., Guo H.B., Liu Y.N., Jiang Y.Y., Dong W.L., He G.X. (2019). Burden of diabetes, hyperglycaemia in China from to 2016: Findings from the 1990 to 2016, global burden of disease study. Diabetes Metab..

[4] Yan Y., Wu T., Zhang M., Li C., Liu Q., Li F. (2022). Prevalence, awareness and control of type 2 diabetes mellitus and risk factors in Chinese elderly population. BMC Public Health.

[5] Shen C., Zhou Z., Lai S., Tao X., Zhao D., Dong W., Li D., Lan X., Gao J. (2019). Urban-rural-specific trend in prevalence of general and central obesity, and association with hypertension in Chinese adults, aged 18–65 years. BMC Public Health.

[6] Zhang S., Sun D., Qian X., Li L., Wu W. (2022). Combined Effects of Obesity and Dyslipidaemia on the Prevalence of Diabetes Amongst Adults Aged ≥ 45 Years: Evidence from a Nationally Representative Cross-Sectional Study. Int. J. Environ. Res. Public Health.

[7] Han C., Liu Y., Sun X., Luo X., Zhang L., Wang B., Ren Y., Zhou J., Zhao Y., Zhang D. (2017). Prediction of a new body shape index and body adiposity estimator for development of type 2 diabetes mellitus: The Rural Chinese Cohort Study. Br. J. Nutr..

[8] Park Y., Kim N.H., Kwon T.Y., Kim S.G. (2018). A novel adiposity index as an integrated predictor of cardiometabolic disease morbidity and mortality. Sci. Rep..

[9] Kim N.H., Park Y., Kim N.H., Kim S.G. (2021). Weight-adjusted waist index reflects fat and muscle mass in the opposite direction in older adults. Age Ageing.

[10] Qin Z., Du D., Li Y., Chang K., Yang Q., Zhang Z., Liao R., Su B. (2022). The association between weight-adjusted-waist index and abdominal aortic calcification in adults aged  ≥  40 years: Results from NHANES 2013–2014. Sci. Rep..

[11] Li Q., Qie R., Qin P., Zhang D., Guo C., Zhou Q., Tian G., Liu D., Chen X., Liu L. (2020). Association of weight-adjusted-waist index with incident hypertension: The Rural Chinese Cohort Study. Nutr. Metab. Cardiovasc. Dis..

[12] Qin Z., Chang K., Yang Q., Yu Q., Liao R., Su B. (2022). The association between weight-adjusted-waist index and increased urinary albumin excretion in adults: A population-based study. Front. Nutr..

[13] Zhao P., Shi W., Shi Y., Xiong Y., Ding C., Song X., Qiu G., Li J., Zhou W., Yu C. (2022). Positive association between weight-adjusted-waist index and hyperuricemia in patients with hypertension: The China H-type hypertension registry study. Front. Endocrinol..

[14] Yu S., Guo X., Yang H., Zheng L., Sun Y. (2014). An update on the prevalence of metabolic syndrome and its associated factors in rural northeast China. BMC Public Health.

[15] Grosso G., Stepaniak U., Micek A., Topor-Mądry R., Pikhart H., Szafraniec K., Pająk A. (2015). Association of daily coffee and tea consumption and metabolic syndrome: Results from the Polish arm of the HAPIEE study. Eur. J. Nutr..

[16] Kim S.M., Lee J.S., Lee J., Na J.K., Han J.H., Yoon D.K., Baik S.H., Choi D.S., Choi K.M. (2006). Prevalence of diabetes and impaired fasting glucose in Korea: Korean National Health and Nutrition Survey 2001. Diabetes Care.

[17] Nguyen N.T., Magno C.P., Lane K.T., Hinojosa M.W., Lane J.S. (2008). Association of hypertension, diabetes, dyslipidemia, and metabolic syndrome with obesity: Findings from the National Health and Nutrition Examination Survey, 1999 to 2004. J. Am. Coll. Surg..

[18] Kim C.S., Ko S.H., Kwon H.S., Kim N.H., Kim J.H., Lim S., Choi S.H., Song K.H., Won J.C., Kim D.J. (2014). Prevalence, awareness, and management of obesity in Korea: Data from the Korea national health and nutrition examination survey (1998–2011). Diabetes Metab. J..

[19] Pi-Sunyer F.X. (2002). The obesity epidemic: Pathophysiology and consequences of obesity. Obes. Res..

[20] Jackson A.S., Stanforth P.R., Gagnon J., Rankinen T., Leon A.S., Rao D.C., Skinner J.S., Bouchard C., Wilmore J.H. (2002). The effect of sex, age and race on estimating percentage body fat from body mass index: The Heritage Family Study. Int. J. Obes. Relat. Metab. Disord..

[21] Lam B.C., Koh G.C., Chen C., Wong M.T., Fallows S.J. (2015). Comparison of Body Mass Index (BMI), Body Adiposity Index (BAI), Waist Circumference (WC), Waist-To-Hip Ratio (WHR) and Waist-To-Height Ratio (WHtR) as predictors of cardiovascular disease risk factors in an adult population in Singapore. PLoS ONE.

[22] Ahn N., Baumeister S.E., Amann U., Rathmann W., Peters A., Huth C., Thorand B., Meisinger C. (2019). Visceral adiposity index (VAI), lipid accumulation product (LAP), and product of triglycerides and glucose (TyG) to discriminate prediabetes and diabetes. Sci. Rep.

[23] Wei J., Liu X., Xue H., Wang Y., Shi Z. (2019). Comparisons of Visceral Adiposity Index, Body Shape Index, Body Mass Index and Waist Circumference and Their Associations with Diabetes Mellitus in Adults. Nutrients.

[24] Ji M., Zhang S., An R. (2018). Effectiveness of A Body Shape Index (ABSI) in predicting chronic diseases and mortality: A systematic review and meta-analysis. Obes. Rev..

[25] Stout M.B., Justice J.N., Nicklas B.J., Kirkland J.L. (2017). Physiological Aging: Links Among Adipose Tissue Dysfunction, Diabetes, and Frailty. Physiology.

[26] Kusminski C.M., Shetty S., Orci L., Unger R.H., Scherer P.E. (2009). Diabetes and apoptosis: Lipotoxicity. Apoptosis.

[27] Arkan M.C., Hevener A.L., Greten F.R., Maeda S., Li Z.W., Long J.M., Wynshaw-Boris A., Poli G., Olefsky J., Karin M. (2005). IKK-beta links inflammation to obesity-induced insulin resistance. Nat. Med..

[28] Hirosumi J., Tuncman G., Chang L., Görgün C.Z., Uysal K.T., Maeda K., Karin M., Hotamisligil G.S. (2002). A central role for JNK in obesity and insulin resistance. Nature.

[29] Nakamura T., Furuhashi M., Li P., Cao H., Tuncman G., Sonenberg N., Gorgun C.Z., Hotamisligil G.S. (2010). Double-stranded RNA-dependent protein kinase links pathogen sensing with stress and metabolic homeostasis. Cell.

[30] Shi H., Kokoeva M.V., Inouye K., Tzameli I., Yin H., Flier J.S. (2006). TLR4 links innate immunity and fatty acid-induced insulin resistance. J. Clin. Investig..

[31] Sun Y., Mu J., Wang D.W., Ouyang N., Xing L., Guo X., Zhao C., Ren G., Ye N., Zhou Y. (2022). A village doctor-led multifaceted intervention for blood pressure control in rural China: An open, cluster randomised trial. Lancet.

[32] Oda E., Kawai R. (2009). Age- and gender-related differences in correlations between abdominal obesity and obesity-related metabolic risk factors in Japanese. Intern. Med..

[33] Szulc P., Duboeuf F., Chapurlat R. (2017). Age-Related Changes in Fat Mass and Distribution in Men-the Cross-Sectional STRAMBO Study. J. Clin. Densitom..

[34] Luo W., Guo Z., Wu M., Hao C., Zhou Z., Yao X. (2015). Interaction of smoking and obesity on type 2 diabetes risk in a Chinese cohort. Physiol. Behav..

